# Development of Fluorescent and Biotin Probes Targeting NLRP3

**DOI:** 10.3389/fchem.2021.642273

**Published:** 2021-04-22

**Authors:** Tim Keuler, Karl Gatterdam, Anil Akbal, Marta Lovotti, Michael Marleaux, Matthias Geyer, Eicke Latz, Michael Gütschow

**Affiliations:** ^1^Pharmaceutical Institute, University of Bonn, Bonn, Germany; ^2^Institute of Structural Biology, University of Bonn, Bonn, Germany; ^3^Institute of Innate Immunity, University of Bonn, Bonn, Germany

**Keywords:** NLRP3, probes, surface plasmon resonance, inflammasome, CRID3, MCC950

## Abstract

Extracellular signals drive the nucleation of the NLRP3 inflammasome which leads to the release of cytokines and causes inflammatory events. Hence, the inflammasome has gained enormous momentum in biomedical basic research. The detailed mechanisms of inflammasome generation and regulation remain to be elucidated. Our study was directed toward the design, convergent synthesis, and initial biochemical evaluation of activity-based probes addressing NLRP3. For this purpose, probes were assembled from a CRID3/MCC950-related NLRP3-binding unit, a linker portion and a coumarin 343 fluorophore or biotin. The affinity of our probes to NLRP3 was demonstrated through SPR measurements and their cellular activity was confirmed by reduction of the interleukin 1β release from stimulated bone marrow-derived macrophages. The initial characterizations of NLRP3-targeting probes highlighted the coumarin probe **2** as a suitable tool compound for the cellular and biochemical analysis of the NLRP3 inflammasome.

## Introduction

**Figure 1:**
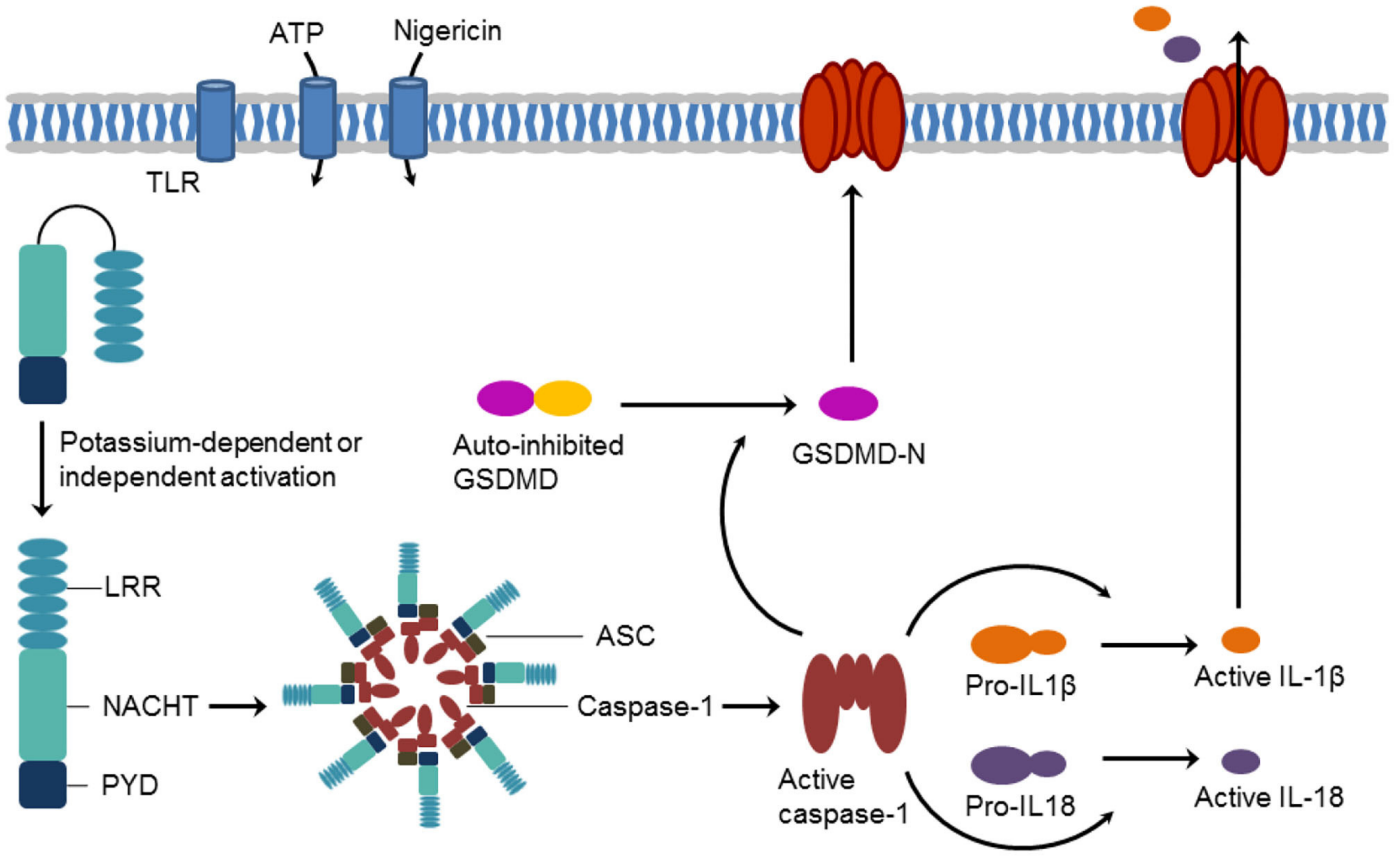
Schematic formation of the NLRP3 inflammasome and induction of pyroptosis.

NLRP3 (NOD-, LRR- and PYD-containing protein 3) has attracted increasing attention as an important player in the (patho)physiology of inflammations. NLRP3 (or NALP3 or cryopyrin) is a tripartite protein of the family of nucleotide-binding oligomerization domain (NOD)-like receptors (NLRs). It contains an N-terminal pyrin domain (PYD), a C-terminal leucine-rich repeat (LRR) domain, and a central NACHT domain. The NACHT domain exists in *NA*IP (neuronal apoptosis inhibitory protein), *C*IITA (MHC class II transcription activator), *H*ET-E (incompatibility locus protein from *Podospora anserine*), and *T*P1 (telomerase-associated protein) proteins and consists itself of four subdomains, i.e., of a nucleotide-binding domain, the helical domain 1, a winged-helix domain, and the helical domain 2 (Eldeeb et al., 2019; Vande Walle et al., 2019). The NLRP3 inflammasome represents a key mediator of the inflammatory response. Inflammasomes are intracellular supramolecular complexes. Their formation is triggered either by damage-associated (such as ATP) or pathogen-associated (such as nigericin) molecular patterns or by Toll-like receptor activation (Baldwin et al., 2016; Chauhan et al., 2020). NLRP3 activation is controlled by PYD and NACHT domain phosphorylation (Song et al., 2017; Stutz et al., 2017). In the NLRP3 inflammasome, NLRP3 acts as a sensor and forms a platform together with the adaptor protein ASC (apoptosis-associated speck-like protein containing a CARD) and the effector protein caspase-1. Crucial steps for NLRP3 inflammasome assembly are NLRP3 oligomerization and recruitment of ASC to NLRP3 oligomers. NLRP3 operates through caspase-1 activation which, in turn, results in the processing of cytokine pro-forms and the release of the maturated pro-inflammatory interleukins IL-1β and IL-18. Caspase 1-catalyzed cleavage of gasdermin D (GSDMD) generates a pore-forming N-terminal protein (GSDMD-N) initiates a lytic form of cell death, referred to as pyroptosis (Figure 1) (Dubois et al., 2019; Pfalzgraff and Weindl, 2019; Broz et al., 2020).

**Figure 2:**
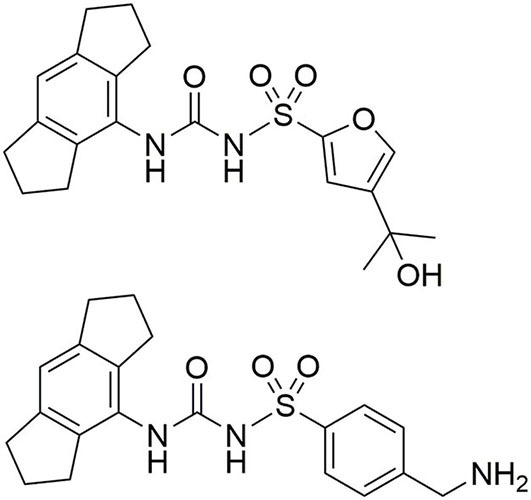
Structure of CRID3 (MCC950, CP-456,773) with a substituted furan moiety (top) and an analog structure with a benzylamine moiety as flexible exit vector (bottom).

NLRP3 is associated with a variety of disorders such as central nervous system (CNS) diseases, rheumatoid arthritis, gout, atherosclerosis, asthma, and crystal nephropathy (Ludwig-Portugall et al., 2016; Dhana et al., 2018; Mangan et al., 2018; Chauhan et al., 2020). The cryopyrin-associated periodic syndromes (CAPS) are autoinflammatory disorders caused by various gain-of-function missense mutations of the NLRP3 gene (Mangan et al., 2018). The strong inflammatory role of NLRP3 provides the impetus for the development of drugs, acting as “NLRP3 inflammasome blockers” (Cocco et al., 2014). On the one hand, agonists could be useful to reverse the immunosuppressive conditions in tumors. On the other hand, inhibitors of the NLRP3 pathway are promising candidates for the treatment of several chronic and auto-inflammatory diseases, including those for which adequate therapies currently do not exist (Mangan et al., 2018). Accordingly, NLRP3 was long considered as a target for the development of small molecule inhibitors. A broad spectrum of NLRP3 inhibitors has been found, from natural products to small synthetic molecules. Cytokine-release-inhibitory-drug-3 (CRID3), a diarylsulfonylurea derivative, showed potent inhibition of the NLRP3 inflammasome formation (Coll et al., 2015). This compound CRID3 is also known as MCC950 or CP-456,773 (Figure 2). Due to reversible binding to the NACHT domain of wildtype NLRP3, ASC oligomerization could be blocked resulting in decreased IL-1β release. It was reported that CRID3/MCC950 binds in proximity to the Walker B motif in NLRP3, impeding the ATP hydrolysis and leading to an inactive NLRP3 conformation (Coll et al., 2019; Tapia-Abellán et al., 2019). Nevertheless, further research attempts are required to decipher NLRP3 inflammasome formation and activation. In this study, we developed new fluorescent and biotin-tagged activity-based probes which could be achieved through a convergent synthetic strategy. These probes are intended to be used, e.g., for fast competition assays, pulldown experiments or confocal microscopy.

## Results and Discussion

Covalent and non-covalent conjugation of proteins to fluorophores is commonly used to study their cellular localization, to discover protein-protein interactions, and to label proteins in their native environment. Fluorescence can be provided by means of a fluorescent low-molecular weight probe possessing sufficient affinity for the target protein. A widely used class of fluorophores, 7-aminocoumarins, are characterized by chemical and enzymatic stability, a small molecular size and large Stokes shifts (Breidenbach et al., 2020). Rigidization of the amino group due to cyclization resulted in increased quantum yields and restored fluorescence in aqueous media. A prominent example is coumarin 343 with an emission maximum at 480 nm in aqueous media after excitation at 440 nm, which is frequently employed to assemble activity-based probes (Terai and Nagano, 2008; Meimetis et al., 2014; Mertens et al., 2014; Kohl et al., 2015). The appendage of a biotin moiety to a bioactive probe offers the opportunity to elucidate and analyze intracellular binding partners of the probe. Biotinylation profits from the exceedingly strong interaction between biotin and either avidin or streptavidin (Trippier, 2013; Verdoes and Verhelst, 2016; Chakrabarty et al., 2019). Accordingly, in order to provide tool compounds to unravel hitherto unknown processes of inflammasome activation and dynamics, we designed NLRP3-specific probes with either coumarin 343 or biotin.

CRID3/MCC950 (Figure 2), the best-known and prevalently applied NLRP3 inflammasome inhibitor, constitutes a sulfonylurea with a hexahydro-*s*-indacen-4-yl group at the terminal nitrogen and a furan moiety at the sulfur atom (Vande Walle et al., 2019; Chauhan et al., 2020; Wu et al., 2020). ^11^C-labeled CRID3/MCC950 was developed for non-invasive PET imaging studies (Hill et al., 2020a). CRID3/MCC950 was chosen as the template for the NLRP3 binding moiety for all of our probes. First, we had to identify an appropriate exit vector to connect either a fluorescent label or a biotin moiety to the NLRP3 ligand without losing entire binding affinity to the target. Recently published structure-activity relationships concerning diarylsulfonylurea-based NLRP3 inhibitors revealed the intact western indacene moiety of CRID3/MCC950 to be crucial for biological activity, and hence for target binding. On the other hand, structural alterations on the eastern sulfonamide part were better tolerated (Hill et al., 2017, 2020b; Agarwal et al., 2020). Two recently developed CRID3/MCC950-derived photoaffinity probes bearing a benzophenone photophore followed the same structural design (Coll et al., 2019; Vande Walle et al., 2019). Similarly, a trifluoromethyl phenyl diazirine photo-cross-linker was introduced at the western part of CRID3/MCC950 whose indacene unit was equipped with a bromine mass tag (Hill et al., 2020b). These photoaffinity probes were employed to covalently label NLRP3 (Coll et al., 2019; Vande Walle et al., 2019).

**Scheme 1:**
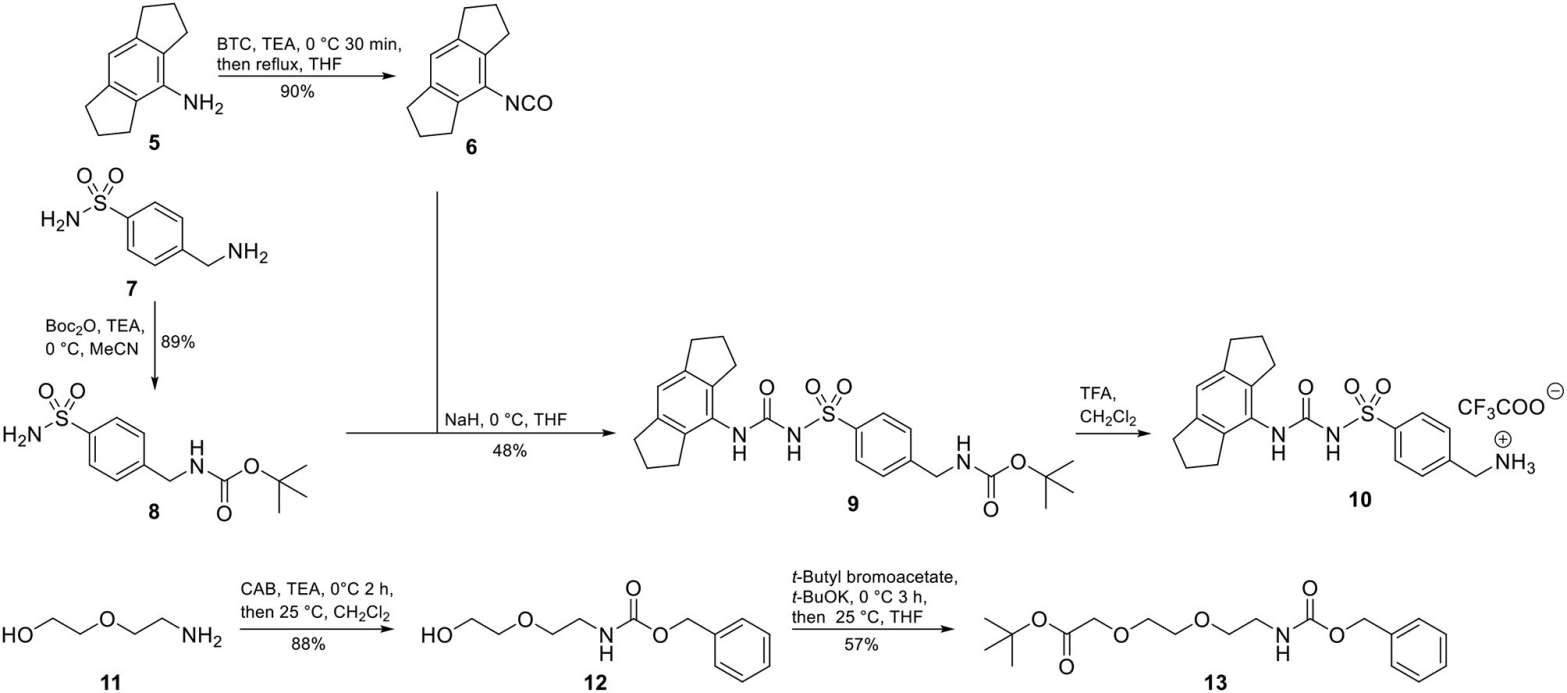
Synthesis of NLRP3 ligand **10** and polyethylene glycol linker **13**.

Accordingly, our molecular design toward NLRP3 probes was based on the introduction of a molecular handle for attaching the label at the eastern part of the diarylsulfonylurea. A network of convergent synthetic routes was employed to assemble the envisaged probes. As depicted in Scheme 1, we synthesized an aromatic sulfonamide (**8**) containing a Boc-protected amino group to be used as exit vector at a later stage of the synthesis. Next, we transformed the hexahydro-*s*-indacen-4-amine **5** in the presence of triphosgene (BTC, [bis(trichloromethyl) carbonate] into the isocyanate **6**. Following the pretreatment of sulfonamide **8** with sodium hydride, the deprotonated intermediate was reacted with isocyanate **6** to obtain the NLRP3 ligand **9**. Compound **9** was deprotected to the trifluoroacetic acid (TFA) salt **10** prior to each coupling reaction.

**Scheme 2:**
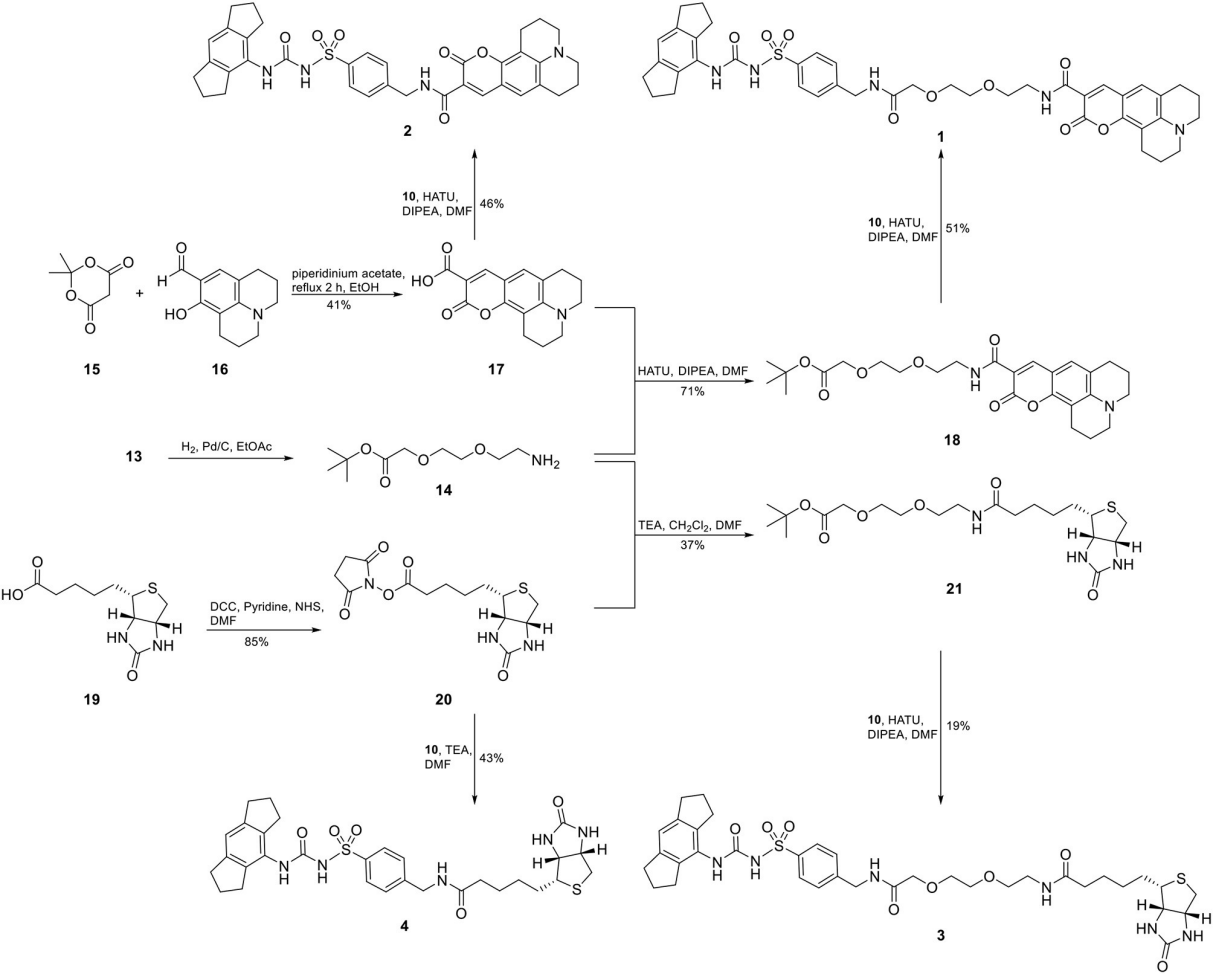
Synthesis of two fluorescent (**1** and **2**) and two biotin-tagged NLRP3 probes (**3** and **4**).

For two final compounds, a short polyethylene glycol linker was chosen to connect the NLRP3 binding moiety and coumarin 343 or biotin. 2-(2-Aminoethoxy)ethanol (**11**) was Cbz-protected and the resulting alcohol **12** was *O*-alkylated with *tert*-butyl bromoacetate to obtain the orthogonally protected linker **13** (Scheme 1), which, in turn, was *N*-deprotected under palladium on carbon (Pd/C)-catalyzed conditions to **14** (Scheme 2).

A Knoevenagel condensation of isopropylidene malonate (**15**) with 8-hydroxyjulolidine-9-carboxaldehyde (**16**) delivered coumarin 343 (**17**) as fluorescent moiety (Scheme 2). HATU-promoted coupling of **17** with the deprotected linker **14** led to compound **18**. After deprotection of **18**, it was coupled with the NLRP3 binding moiety **10** to obtain the fluorescent NLRP3 probe **1**. For a shorter coumarin-labeled probe, coumarin 343 (**17**) was directly connected to the NLRP3 ligand **10** to achieve probe **2**. To obtain the biotin-tagged probes, we first activated D-biotin with *N*-hydroxysuccinimid (NHS) to the active ester **20**. This was reacted with the deprotected linker **14**. After deprotection of **21**, a further HATU coupling was performed to yield the biotin-tagged probe **3**. Direct linkage of **20** with the NLRP3 ligand **10** delivered the short biotin-tagged probe **4**.

**Figure 3:**
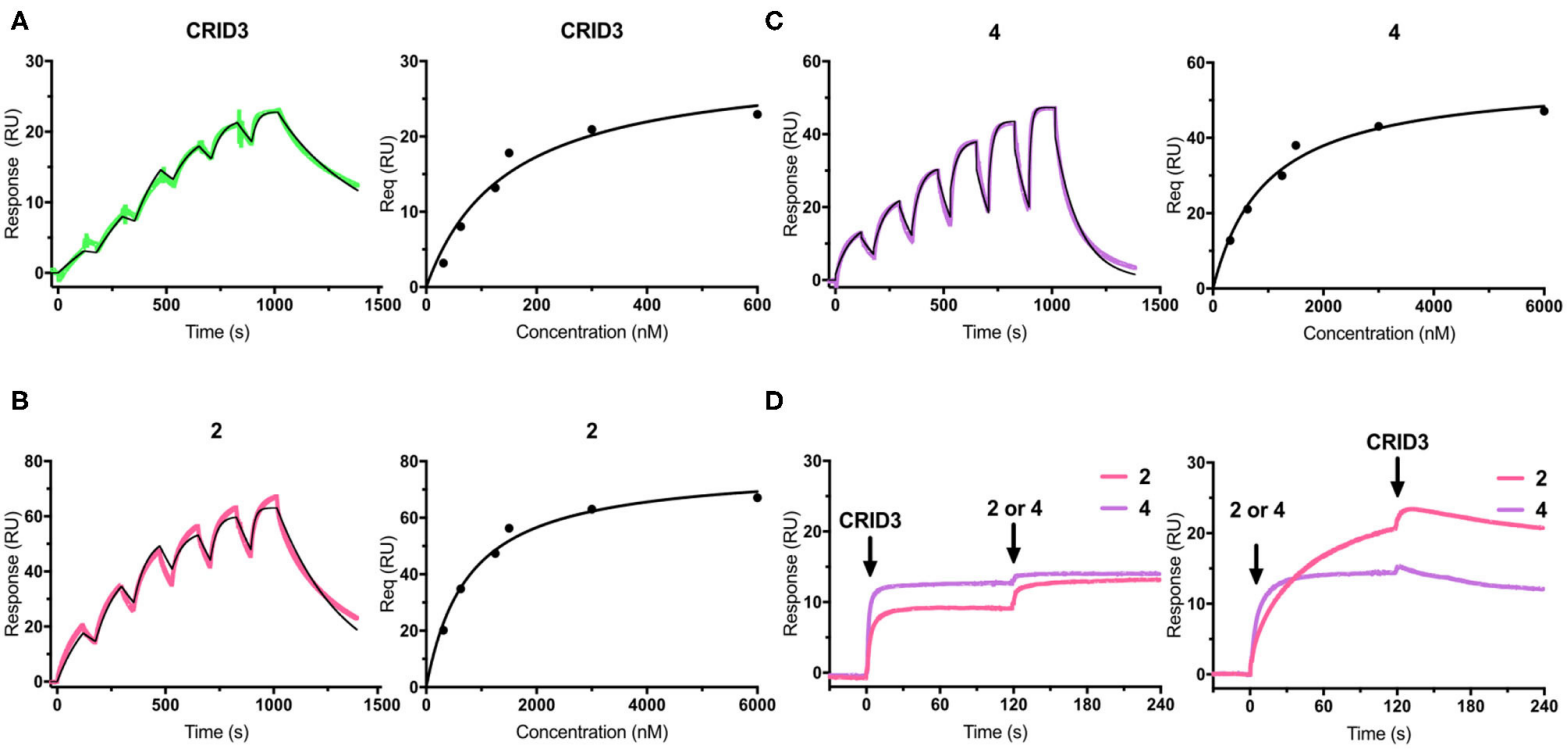
**(A**–**C)** SPR sensorgrams (left) and concentration-binding response unit curves (right) of CRID3/MCC950 **(A)**, compound **2 (B)**, and compound **4 (C)** injected on the chip surface loaded with human NLRP3-PYD-NACHT. The binding parameters obtained from the sensorgrams are listed in Table 1. Data were fitted to a 1:1 binding model. **(D)** SPR sensorgrams of CRID3/MCC950, compound **2**, and compound **4** injected in competition mode on the chip surface loaded with human NLRP3-PYD-NACHT. Either CRID3/MCC950 (left) or compound **2** or **4** (right) were provided in the first injection step, followed by a second injection step after 120 s, where equimolar concentrations of the second compound were injected in presence of the first compound.

Surface plasmon resonance (SPR) is a powerful and versatile spectroscopic method, but less commonly used for the analysis of low-molecular weight probes for functional proteins. SPR spectroscopy allows a real-time measurement and the label-free detection of biomolecular interactions through a surface-sensitive response. In order to assess the suitability of our probes, they were subjected to an SPR analysis and compounds **1**–**4**, together with CRID3/MCC950, were analyzed for their binding behavior to human NLRP3. For that purpose, recombinant biotinylated human NLRP3-PYD-NACHT domain protein derived from HEK293T cells was immobilized on a streptavidin-functionalized sensor chip (sensor chip SA). After a stabilization period, the analytes, CRID3/MCC950 and the respective test compound, were injected on single channels using single-cycle kinetics (Figure 3 for compounds **2** and **4**; for compounds **1** and **3**, see Supplementary Figure 1). The SPR data (Table 1) revealed a dissociation constant *K*_D_ of 322 nM for the fluorescently labeled compound **2** and a *K*_D_ value of 31 nM for CRID3/MCC950. The dissociation constant *K*_D_ for the fluorescently labeled probe with the PEG linker (**1**) was determined to be 813 nM, whereas the two biotinylated probes (**3**, **4**) bound with *K*_D_ values of 1,030 and 611 nM, respectively. These data confirm that the molecular probes **1**–**4** attached with fluorescent or biotin labels to the scaffold of CRID3/MCC950 bind to recombinant NLRP3 protein. The smaller compounds (**2** and **4**) exhibited lower dissociation constants than their linker-connected counterparts (**1** and **3**). To confirm that compound **2** and **4** have the same binding site as CRID3/MCC950, we injected two ligands 120 s after the first injection (Figure 3D). No significant response change, indicative of a second binding site, was observed in either succession of the injections (left and right), which points to the assumption that CRID3/MCC950 and both compounds interact with the same binding site on the target protein NLRP3. Although CRID3/MCC950 possessed a higher affinity to NLRP3, our compounds bound sufficiently tight, making them promising candidates for a further characterization as suitable NLRP3 probes. However, the reversible binding mode may obstruct the use for Western blotting analysis.

**Table 1:** Second-order on-rate constants for association (*k*_a_), first-order off-rate constants for dissociation (*k*_d_), and kinetic dissociation constants (*K*_D_) for NLRP3 ligands.

**Compound**	***k*_**a**_ (M^**−1**^s^**−1**^)**	***k*_**d**_ (s^**−1**^)**	***K*_**D**_ (nM)**
CRID3/MCC950	7.5 × 10^4^	2.3 × 10^−3^	31
**1**	1.3 × 10^4^	1.0 × 10^−2^	813
**2**	1.0 × 10^4^	3.3 × 10^−3^	322
**3**	2.2 × 10^4^	2.2 × 10^−2^	1,030
**4**	1.4 × 10^4^	8.8 × 10^−3^	611

**Figure 4:**
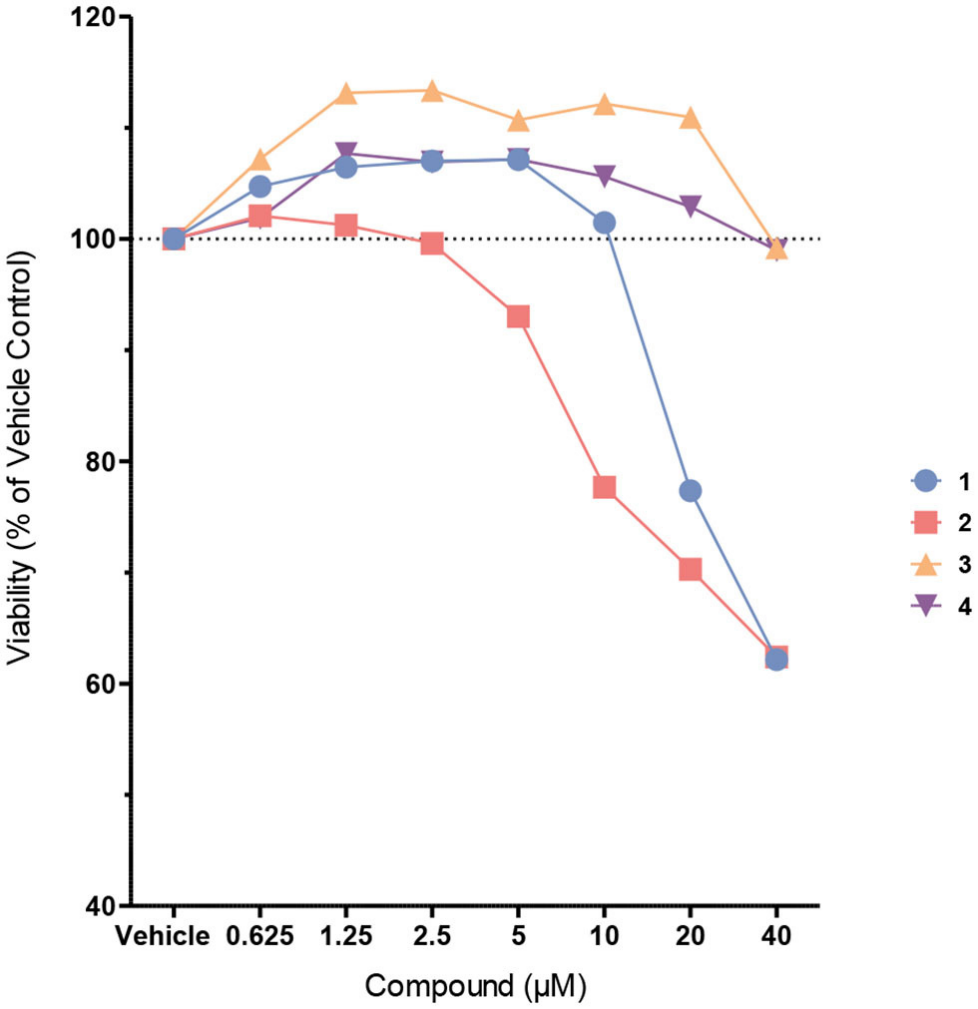
Cell viability of immortalized murine bone marrow-derived macrophages after treatment with probes **1**–**4**. Cells were treated with the probes at different concentrations in Opti-MEM for 4 h. Cell viability was assessed using the CellTiter-Blue cell viability assay.

Next, the probes were examined in cell culture experiments. First, we determined the cytotoxicity of probes **1**–**4** at different concentrations between 625 nM and 40 μM in immortalized murine bone marrow-derived macrophages (Figure 4). While biotin probes **3** and **4** were proved to be non-toxic up to 40 μM, and the coumarin probe **1** was non-toxic up to 10 μM, the coumarin probe **2** exhibited some cytotoxicity at concentrations higher than 2.5 μM.

**Figure 5:**
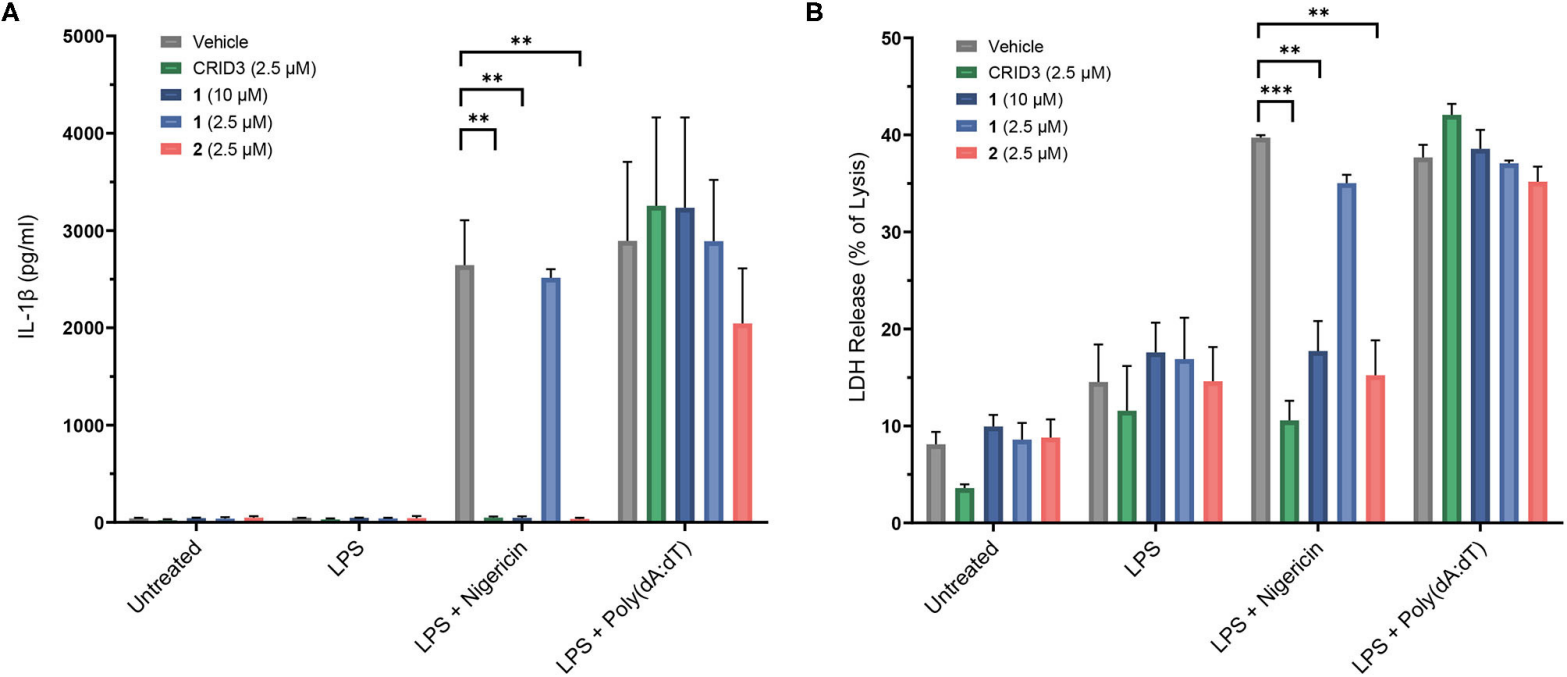
Inhibition of IL-1β release **(A)** in immortalized murine bone marrow-derived macrophages after treatment with CRID3/MCC950 and fluorescent probes **1** and **2**. LDH release **(B)** of immortalized murine bone marrow-derived macrophages after treatment with CRID3/MCC950 and fluorescent probes **1** and **2**. All data are means ± SEM (*n* = 3), Student's *t*-test. *P ≤ 0.05, **P ≤ 0.01, ***P ≤ 0.001.

The following experiments were performed with probes **1**–**4** in immortalized murine bone marrow-derived macrophages. Nigericin, an antibiotic carboxylate ionophore, was used as the activator of the NLRP3 inflammasome, owing to its ability to induce a potassium efflux. In addition, we addressed the absent in melanoma 2 (AIM2) inflammasome, a distinct inflammasome complex, which, upon assembly, can also activate caspase-1 and cause cells to release IL-1β (Hornung et al., 2009). For this purpose, AIM2 was stimulated by transfection with poly(deoxyadenylic-deoxythymidylic) acid [poly(dA:dT)] after pretreatment with LPS. A Homogeneous Time Resolved Fluorescence (HTRF) assay was employed to determine the release of IL-1β (Figure 5). Probe **1** was used at 2.5 μM and 10 μM, i.e., with concentrations found to be non-toxic in the same cells. We observed a complete inhibition of IL-1β release at a concentration of 10 μM, but not at 2.5 μM. Probe **2** was only investigated at 2.5 μM due to toxic effects at higher concentration. Notably, this probe showed complete inhibition at 2.5 μM, just like CRID3/MCC950 at this concentration. The biotin-tagged probes **3** and **4**, at 20 μM each, did not significantly affect the cytokine release (see Supplementary Figure 2). For reasons to be clarified, these cellular data only partially agree with the affinity of the entire subset of compounds **1**-**4** for NLRP3 as determined in the SPR assay. The coumarin-containing compound **2** at 20 μM also reduced AIM2-dependent IL-1β release initiated by poly(dA:dT) (data not shown), obviously due to cytotoxicity. However, at 2.5 μM, only a slightly reduced AIM2-dependent release of IL-1β was observed (Figure 5). As expected, CRID3/MCC950 and probe **1** did not affect this process.

To assess pyroptosis, cytosolic lactate dehydrogenase (LDH) release was measured as a marker of cell lysis. In contrast to priming with lipopolysaccharide (LPS) alone, priming with LPS followed by treatment with nigericin and poly(dA:dT), respectively, clearly increased cell lysis. CRID3/MCC950 and probe **2**, each at a concentration of 2.5 μM, as well as probe **1** at 10 μM prevented the NLRP3-dependent, but not the AIM2-dependent cell death (Figure 5). This rescue effect can be attributed to the anti-pyroptotic activity of the compounds and was in agreement with the inhibitory potency of the investigated compounds regarding IL-1β release.

**Figure 6:**
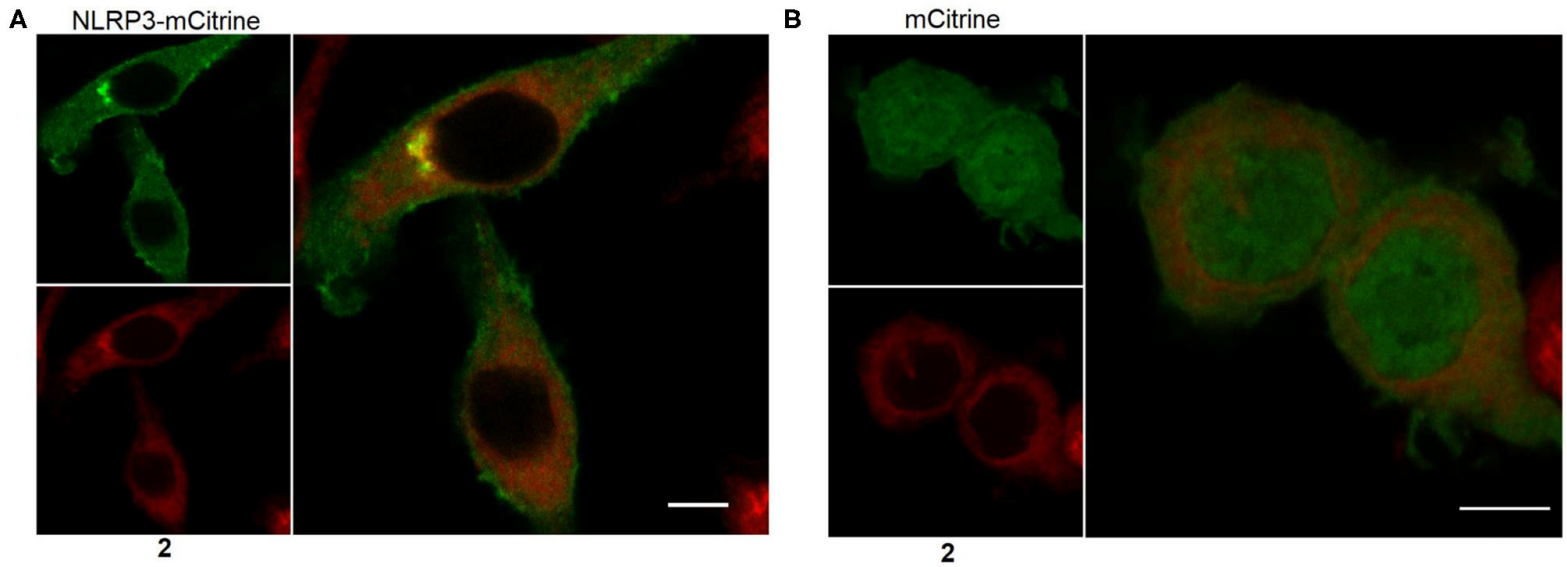
Representative microscopic images showing NLRP3 in immortalized murine macrophages overexpressing NLRP3 fused to mCitrine **(A)** or overexpressing mCitrine **(B)**. Cells were treated with 5 μM of probe **2** for 30 min. Wavelengths for excitation / emission were as follows 516 nm / 529 nm for mCitrine and 393 nm / 509 nm for coumarin 343. The experiment was performed three times. The bars indicate 5 μm.

The most suitable probe, **2**, was subjected to confocal microscopy for detection of NLRP3 in non-stimulated, immortalized murine macrophages overexpressing NLRP3 fused to mCitrine, a monomeric variant of the yellow fluorescent protein. The confocal images show that NLRP3 staining was colocalized with the yellow fluorescence (Figure 6A). On the other hand, the presence of the NLRP3 fusion protein was a prerequisite for colocalization as shown in the negative control (Figure 6B). This incipient result indicated a successful application of this coumarin-labeled NLRP3 ligand for future studies.

In conclusion, we designed and synthesized four probes based on the structure of the prototypical NLRP3 inhibitor CRID3/MCC950. An appropriate exit vector was established for the attachment of a fluorescent or biotin label. All of our probes were demonstrated to bind to the NLRP3 protein. Two coumarin-labeled probes were shown to reach the cytosolic target and to block the final cellular response. One of them, probe **2**, is expected to serve as an appropriate tool compound in the field of inflammasome research which is currently ongoing in our laboratories.

## Experimental Section

### General Experimental Procedures

Chemicals were purchased from ABCR, BLDpharm, Fisher Chemical, Sigma Aldrich and Tokyo Chemical Industry. Thin layer chromatography was carried out with pre-coated silica gel (60 F254) aluminum sheets from Merck. Detection was performed with UV light at 254 and 360 nm or with AgNO_3_ staining. Acros Organics silica gel 60 (70–230 mesh) was taken for preparative column chromatography. Preparative silica gel flash column chromatography was performed on an Interchim Puriflash PF420 system with diode-array detection (DAD) from 200 to 400 nm. Melting points were measured on a Büchi 510 oil bath apparatus. HR-ESI-MS were recorded on a Bruker micrOTOF-Q mass spectrometer coupled with a HPLC Dionex UltiMate 3000 or a LTQ Orbitrap XL. ESI-MS mass spectra were recorded on an API2000 mass spectrometer coupled with an Agilent HPLC HP1100 using an EC50/2 Nucleodur C18 Gravity 3 μm column. The purity of synthesized compounds was determined by HPLC-DAD obtained on an Agilent HP1100 LC-MS instrument and the purity of all final compounds was confirmed to be >95% (HPLC-DAD). NMR spectra were recorded on a Bruker Avance DRX 500 (500 MHz ^1^H-NMR, 126 MHz ^13^C-NMR) and a Bruker Avance III 600 (600 MHz ^1^H-NMR, 151 MHz ^13^C-NMR). Chemical shifts are given in parts per million (ppm) referring to the signal center using the solvent peaks for reference: DMSO-*d*_6_ (2.49/39.7).

### Syntheses

#### 4-Isocyanato-1,2,3,5,6,7-hexahydro-*s*-indacene (6)

Triphosgene (BTC; 0.53 g, 1.8 mmol) was dissolved in dry THF (30 mL) at 0 °C under nitrogen atmosphere. The solution was stirred for 30 min at 0 °C. Triethylamine (TEA; 0.36 g, 3.6 mmol) was added in one portion and a solution of 1,2,3,5,6,7-hexahydro-*s*-indacen-4-amine (**5**, 0.31 g, 1.8 mmol) in dry THF (30 mL) was added dropwise over 30 min. The reaction mixture was refluxed for 30 min. All volatiles were evaporated. The residue was taken up in dry THF (30 mL), filtered and concentrated to yield a crude brown oil, which was directly used in the next step without further purification. Yield 90%; *R*_f_ = 0.67 (hexane).

#### *tert*-Butyl (4-Sulfamoylbenzyl)carbamate (8)

4-Aminomethylbenzenesulfonamide × HCl (**7**, 2.23 g, 10 mmol) was dissolved in MeCN (40 mL) and cooled to 0°C. TEA (2.02 g, 20 mmol) was added and the solution was stirred for 5 min. Di-*tert*-butyl dicarbonate (Boc_2_O; 2.18 g, 10 mmol) was dissolved in MeCN (10 mL) and added dropwise to the reaction mixture which was allowed to stir overnight at room temperature. The volatiles were evaporated and the residue was taken up in EtOAc (60 mL). The organic layer was washed with H_2_O (3 × 60 mL) and brine (60 mL), dried over Na_2_SO_4_, filtered and concentrated to yield the product as a white solid. Yield 89%; mp: 144-146 °C; *R*_f_ = 0.42 (petroleum ether/EtOAc 1+1); ^1^H NMR (600 MHz, DMSO-*d*_6_) δ 1.39 (s, 9H), 4.18 (d, *J* = 6.1 Hz, 2H), 7.28 (s, 2H), 7.40 (d, *J* = 8.0 Hz, 2H), 7.46 (t, *J* = 6.2 Hz, 1H), 7.76 (d, *J* = 8.2 Hz, 2H); ^13^C NMR (151 MHz, DMSO-*d*_6_) δ 28.20, 43.05, 77.98, 125.64, 127.17, 142.50, 144.23, 155.77; LC-MS (ESI) (90% H_2_O to 100% MeOH in 10 min, then 100% MeOH to 20 min, DAD 220-400 nm), *t*_R_= 8.45 min, 100% purity, *m/z* [M+H]^+^ calcd for C_12_H_18_N_2_O_4_S 287.1, found 287.0.

#### *tert*-Butyl (4-(*N*-([1,2,3,5,6,7-Hexahydro-*s*-indacen-4-yl]carbamoyl)sulfamoyl)benzyl)carbamate (9)

Compound **8** (0.46 g, 1.6 mmol) was dissolved in dry THF (15 mL). NaH (60% dispersion in mineral oil; 0.12 g, 2.9 mmol) was added in one portion and the reaction mixture was stirred at 0 °C under nitrogen atmosphere for 30 min. A solution of compound **6** (0.32 g, 1.6 mmol) in dry THF (10 mL) was added and the reaction mixture was allowed to stir at room temperature under nitrogen atmosphere for 4 h. All volatiles were evaporated. The solid residue was taken up in water (10 mL). The solution was acidified with 2 N HCl and precipitate formed was filtered off, washed with EtOAc (10 mL) and dried under high vacuum to yield a white solid. Yield 48%; mp: >250°C (decomposition); *R*_f_ = 0.55 (petroleum ether/EtOAc 1+1); ^1^H NMR (600 MHz, DMSO-*d*_6_) δ 0.57 (s, 9H), 1.04–1.11 (m, 4H), 1.68–1.69 (m, 2H), 1.82 (t, *J* = 7.4 Hz, 4H), 1.92 (t, *J* = 7.4 Hz, 4H), 5.94 (s, 1H), 6.40 (d, *J* = 7.9 Hz, 2H), 6.57 (t, *J* = 6.1 Hz, 1H), 6.63 (s, 1H), 6.88 (d, *J* = 8.0 Hz, 2H), one NH signal is missing due to proton exchange; ^13^C NMR (151 MHz, DMSO-*d*_6_) δ 25.04, 28.21, 30.48, 32.56, 43.10, 77.78, 115.49, 126.01, 126.04, 132.66, 136.62, 141.37, 142.06, 145.95, 155.74, 158.43; LC-MS (ESI) (90% H_2_O to 100% MeOH in 10 min, then 100% MeOH to 20 min, DAD 220-400 nm), *t*_R_= 11.20 min, 100% purity, *m/z* [M+H]^+^ calcd for C_15_H_31_N_3_O_5_S 486.2, found 486.4.

#### *tert*-Butyl 2-(2-(2-(11-Oxo-2,3,6,7-tetrahydro-1*H*,5*H*,11*H*-pyrano[2,3-*f*]pyrido[3,2,1-*ij*]quinoline-10-carboxamido)ethoxy)ethoxy)acetate (18) (Kohl et al., 2015)

The orthogonal protected linker *tert*-butyl 3-oxo-1-phenyl-2,7,10-trioxa-4-azadodecan-12-oate (**13**, 0.353 g, 1 mmol; see Supplementary Material) was dissolved in dry EtOAc (10 mL) and treated with 10% Pd/C. The suspension was stirred under H_2_ (1 atm, balloon) overnight. The mixture was filtered through celite and the filtrate was concentrated to yield the deprotected linker **14**. 11-Oxo-2,3,6,7-tetrahydro-1*H*,5*H*,11*H*-pyrano[2,3-*f* ]pyrido[3,2,1-*ij*]quinoline-10-carboxylic acid (**17**, 0.285 g, 1 mmol; see Supplementary Material) was dissolved in dry DMF (5 mL). HATU (0.418 g, 1.1 mmol) and DIPEA (0.388 g, 3 mmol) were added. The mixture was stirred for 30 min under argon atmosphere. Then, the deprotected linker **14**, dissolved in DMF (5 mL) was added. The reaction mixture was stirred for 4 h at room temperature. The solution was concentrated under high vacuum. The crude product was purified via silica gel flash column chromatography using a gradient from petroleum ether (100%) to EtOAc (100%) to yield an orange oil. Yield 71%; *R*_f_ = 0.43 (EtOAc); ^1^H NMR (500 MHz, DMSO-*d*_6_) δ 1.41 (s, 9H), 1.84–1.92 (m, 4H), 2.69–2.76 (m, 4H), 3.31–3.35 (m, 4H), 3.44–3.48 (m, 2H), 3.52–3.55 (m, 2H), 3.55–3.61 (m, 4H), 3.99 (s, 2H), 7.25 (s, 1H), 8.52 (s, 1H), 8.78 (t, *J* = 5.5 Hz, 1H); ^13^C NMR (126 MHz, DMSO-*d*_6_) δ 19.55 (two carbons), 20.50, 26.76, 27.69, 38.74, 48.96, 49.49, 68.16, 69.01, 69.53, 69.83, 80.54, 104.59, 107.32, 107.81, 119.39, 127.10, 147.48, 147.97, 152.05, 161.79, 162.41, 169.28; LC-MS (ESI) (90% H_2_O to 100% MeOH in 10 min, then 100% MeOH to 20 min, DAD 220-500 nm), *t*_R_= 11,70 min, 94% purity, *m/z* [M+H]^+^ calcd for C_26_H_34_N_2_O_7_ 487.2, found 487.3.

#### *N*-(2-(2-(2-([4-(*N*-([1,2,3,5,6,7-Hexahydro-*s*-indacen-4-yl]carbamoyl)sulfamoyl)benzyl]amino)-2-oxoethoxy)ethoxy)ethyl)-11-oxo-2,3,6,7-tetrahydro-1*H*,5*H*,11*H*-pyrano[2,3-*f*]pyrido[3,2,1-*ij*]quinoline-10-carboxamide (1)

Compound **18** (0.15 g, 0.3 mmol) was dissolved in dry CH_2_Cl_2_ (3 mL) and trifluoroacetic acid (TFA; 3 mL) was added. The solution was stirred for 2 h. After evaporation of all volatiles, the deprotected acid compound was dried under high vacuum. Compound **9** (0.15 g, 0.3 mmol) was dissolved in a mixture of dry CH_2_Cl_2_ (3 mL) and TFA (3 mL) and allowed to stir 2 h. After evaporation of all volatiles, the resulting trifluoroacetate salt **10** was dried under high vacuum. The deprotected acid compound was dissolved in dry DMF (5 mL). Under argon atmosphere, HATU (0.13 g, 0.33 mmol), DIPEA (0.12 g, 0.9 mmol) and a solution of the trifluoroacetate salt **10** in dry DMF (5 mL) were added. It was allowed to stir for 4 h, before it was evaporated under high vacuum. The crude product was purified via silica gel flash column chromatography with a gradient from CH_2_Cl_2_ to CH_2_Cl_2_/MeOH (8+2) to yield a yellow solid. Yield 51%; mp: 150-152°C; *R*_f_ = 0.45 (CH_2_Cl_2_/MeOH 9+1); ^1^H NMR (600 MHz, DMSO-*d*_6_) δ 1. –1.92 (m, 8H), 2.51–2.54 (m, 4H, signal is mainly obscured by DMSO signal), 2.65 (t, *J* = 6.4 Hz, 2H), 2.69 (t, *J* = 6.2 Hz, 2H), 2.74 (t, *J* = 7.4 Hz, 4H), 3.27–3.29 (m, 4H, signal is mainly obscured by H_2_O signal), 3.44–3.47 (m, 2H), 3.52–3.56 (m, 2H), 3.60–3.65 (m, 4H), 3.97 (s, 2H), 4.42 (d, *J* = 6.2 Hz, 2H), 6.90 (s, 1H), 7.22 (s, 1H), 7.48 (d, 2H), 7.87 (d, 2H), 8.05 (s, 1H), 8.31 (t, *J* = 6.3 Hz, 1H), 8.50 (s, 1H), 8.83 (t, *J* = 5.5 Hz, 1H), 10.70 (s, 1H); ^13^C NMR (151 MHz, DMSO-*d*_6_) δ 19.48, 19.51, 20.49, 24.94, 26.77, 30.04, 32.38, 38.69, 41.37, 48.93, 49.50, 69.00, 69.33, 70.03, 70.26, 104.58, 107.36, 107.71, 117.90, 119.43, 127.10, 127.23, 127.43, 128.58, 137.10, 138.44, 143.01, 145.17, 147.52, 147.99, 149.02, 152.04, 161.97, 162.41, 169.45; LC-MS (ESI) (90% H_2_O to 100% MeOH in 10 min, then 100% MeOH to 20 min, DAD 220-500 nm), *t*_R_= 11,68 min, 97% purity, *m/z* calcd for C_42_H_47_N_5_O_9_S 798.3, found 798.7; HRMS(ESI) *m/z* [M+Na]^+^ calcd 820.2987, found 820.2990.

#### *tert*-Butyl 2-(2-(2-(5-([3a*R*,4*R*,6a*S*]-2-Oxohexahydro-1*H*-thieno[3,4-*d*]imidazol-4-yl)pentanamido)ethoxy)ethoxy)acetate (21)

The orthogonal protected linker *tert*-butyl 3-oxo-1-phenyl-2,7,10-trioxa-4-azadodecan-12-oate (**13**, 0.53 g, 1.5 mmol; see Supplementary Material) was dissolved in dry EtOH (10 mL) and treated with 10% Pd/C. The reaction mixture was stirred under H_2_ (1 atm, balloon) overnight. The mixture was filtered through celite and the filtrate was concentrated to yield the deprotected linker **14**. 2,5-Dioxopyrrolidin-1-yl 5-([3a*R*,4*R*,6a*S*]-2-oxohexahydro-1*H*-thieno[3,4-*d*]imidazol-4-yl)pentanoate (**20**, 0.51 g, 1.5 mmol; see Supplementary Material) was dissolved in dry DMF (5 mL) and dry CH_2_Cl_2_ (5 mL) under argon atmosphere. TEA (0.46 g, 4.5 mmol) and the deprotected linker **14**, dissolved in DMF (5 mL) were added. The combined mixture was stirred overnight at room temperature. The solution was evaporated under high vacuum. The crude product was purified via silica gel column chromatography using CH_2_Cl_2_/MeOH (9+1) as eluent to yield a colorless resin. Residues of *N*-hydroxysuccinimide (NHS) were removed in the next step. Yield 37%; *R*_f_ = 0.23 (CH_2_Cl_2_/MeOH 9+1); ^1^H NMR (500 MHz, DMSO-*d*_6_) δ 1.24–1.35 (m, 2H), 1.42 (s, 9H), 1.45–1.56 (m, 3H), 1.56–1.67 (m, 1H), 2.06 (t, *J* = 7.3 Hz, 2H), 2.60–2.62 (m, 1H, signal is mainly obscured by NHS signal), 2.82 (dd, *J* = 12.4, 5.2 Hz, 1H), 3.07–3.12 (m, 1H), 3.17–3.20 (m, 2H), 3.39–3.41 (m, 2H), 3.51 – 3.58 (m, 4H), 3.97 – 3.98 (m, 2H), 4.10 – 4.16 (m, 1H), 4.29 – 4.32 (m, 1H), 6.32 (s, 1H), 6.38 (s, 1H), 7.78 (t, *J* = 5.5 Hz, 1H); ^13^C NMR (126 MHz, DMSO-*d*_6_) δ 25.18, 27.74, 27.99, 28.14, 35.06, 38.41, 55.36, 59.17, 61.00, 68.11, 69.10, 69.42, 69.82, 80.62, 162.64, 172.07, 172.67. One signal is missing solvent peak); LC-MS (ESI) (90% H_2_O to 100% MeOH in 10 min, then 100% MeOH to 20 min, DAD 200-400 nm), *t*_R_= 9.19 min, *m/z* [M+H]^+^ calcd for C_20_H_35_N_3_O_6_S 446.2, found 446.2.

#### *N*-(2-(2-(2-([4-(*N*-([1,2,3,5,6,7-Hexahydro-*s*-indacen-4-yl]carbamoyl)sulfamoyl)benzyl]amino)-2-oxoethoxy)ethoxy)ethyl)-5-([3a*R*,4*R*,6a*S*]-2-oxohexahydro-1*H*-thieno[3,4-*d*]imidazol-4-yl)pentanamide (3)

Compound **21** (0.27 g, 0.6 mmol) was dissolved in dry CH_2_Cl_2_ (5 mL) and TFA (5 mL) was added. The solution was stirred for 2 h. After evaporation of all volatiles, the deprotected acid compound was dried under high vacuum. Compound **9** (0.29 g, 0.6 mmol) was dissolved in a mixture of dry CH_2_Cl_2_ (5 mL) and TFA (5 mL) and allowed to stir 2 h. After evaporation of all volatiles, the resulting trifluoroacetate salt **10** was dried under high vacuum. The deprotected acid compound was dissolved in dry DMF (5 mL). Under argon atmosphere, HATU (0.25 g, 0.66 mmol), DIPEA (0.23 g, 1.8 mmol) and a solution of the trifluoroacetate salt **10** in dry DMF (5 mL) were added. It was allowed to stir for 4 h, before it was evaporated under high vacuum. The crude product was purified via silica gel flash column chromatography with a gradient from CH_2_Cl_2_ to CH_2_Cl_2_/MeOH (8+2) to yield a white solid. Yield 19%; mp: 140–142°C; *R*_f_ = 0.20 (CH_2_Cl_2_/MeOH 9+1); ^1^H NMR (600 MHz, DMSO-*d*_6_) δ 1.24–1.32 (m, 2H), 1.42–1.53 (m, 3H), 1.57–1.63 (m, 1H), 1.92 (p, *J* = 7.4 Hz, 4H), 2.05 (t, *J* = 7.4 Hz, 2H), 2.54 (t, *J* = 7.4 Hz, 4H), 2.57 (d, *J* = 12.3 Hz, 1H), 2.77 (t, *J* = 7.4 Hz, 4H), 2.81 (dd, *J* = 12.4, 5.1 Hz, 1H), 3.05–3.10 (m, 1H), 3.18 (q, *J* = 5.8 Hz, 2H), 3.40 (t, *J* = 5.9 Hz, 2H), 3.54–3.63 (m, 4H), 3.96 (s, 2H), 4.10–4.13 (m, 1H), 4.28 – 4.31 (m, 1H), 4.39 (d, *J* = 6.2 Hz, 2H), 6.33 (s, 1H), 6.38 (s, 1H), 6.92 (s, 1H), 7.47 (d, *J* = 8.2 Hz, 2H), 7.80 (t, *J* = 5.7 Hz, 1H), 7.87 (d, 2H), 8.06 (s, 1H), 8.36 (t, *J* = 6.3 Hz, 1H), 10.71 (s, 1H); ^13^C NMR (151 MHz, DMSO-*d*_6_) δ 24.98, 25.21, 28.00, 28.15, 30.07, 32.41, 35.06, 38.38, 41.36, 55.37, 59.17, 61.00, 69.14, 69.33, 70.00, 70.26, 117.86, 127.23, 127.49, 128.74, 137.11, 138.75, 143.01, 145.00, 149.35, 162.66, 169.53, 172.13, One signal is missing (overlapping solvent peak); LC-MS (ESI) (90% H_2_O to 100% MeOH in 10 min, then 100% MeOH to 20 min, DAD 220-400 nm), *t*_R_= 9,86 min, 96% purity, *m/z* [M+H]^+^ calcd for C_36_H_48_N_6_O_8_S_2_ 757.3, found 757.9; HRMS (ESI) *m/z* [M+H]^+^ calcd 757.3048, found 757.3049.

### Biochemical and Cell Biological Procedures

#### Expression of Recombinant NLRP3 Protein

HEK293T cells were transiently co-transfected with human avi-FLAG-His10-NLRP3-PYD-NACHT domain (pIRES puro 3 vector) and BirA (pcDNA3.1 vector) for in-cell biotinylation at the lysine amino group of the Avi-tag. After 24 h expression, cells were chemically disrupted and avi-FLAG-His_10_-NLRP3-PYD-NACHT domain was isolated using anti-FLAG M2 affinity beads (Sigma Aldrich). Eluted protein was stored snap frozen in liquid nitrogen, aliquoted, and stored at −80 °C.

#### Surface Plasmon Resonance Spectroscopy

SPR experiments were performed using a Biacore 8K instrument (GE Healthcare). The flow system was cleaned using the maintenance “Desorb” function (Desorb Kit, GE Healthcare). The system was flushed with running buffer (10 mM HEPES pH 7.4, 200 mM NaCl, 0.5 mM ADP, 0.5 mM tris(2-carboxyethyl)phosphine (TCEP), 2 mM MgCl_2_, 1 g/L carboxymethyl dextran hydrogel (CMD), 0.05% Tween20, 2% DMSO) and all steps were performed at 25 °C chip temperature. Before immobilization, a streptavidin functionalized sensor chip (Series S Sensor Chip SA, GE Healthcare) was conditioned with three consecutive 1-min injections of 1 M NaCl in 50 mM NaOH (10 μL/min). Biotinylated NLRP3-PYD-NACHT protein (2 μL/min, 900 s) was immobilized on the sensor chip flow cell 2. The flow system was washed using 50% isopropanol in 1 M NaCl and 50 mM NaOH. Free streptavidin binding sites were blocked by four consecutive 2-min injections (1,000 nM, 10 μL/min) of Biotin-PEG (Mn = 2,300 Da) (Sigma Aldrich). For kinetic binding measurements, different concentrations of CRID3/MCC950 (31.25 nM, 62.5 nM, 125 nM, 150 nM, 300 nM, 600 nM) or test compounds **1**–**4** (312.5 nM, 625 nM, 1,250 nM, 1,500 nM, 3,000 nM, 6,000 nM) were injected (30 μL/min, association: 120 s, dissociation: 60/360 s) over both flow cells. For competition measurements, the first compound was injected (30 μl/min, monitored over 120 s) at a concentration 10-times the kinetic *K*_D_ of test compound **2** or **4**, respectively. In a second injection step, equimolar concentration of the second compound was injected (30 μl/min, 120 s) in presence of the first compound. Data were collected at a rate of 10 Hz. The binding data were double referenced by blank cycle and reference flow cell subtraction. Data were corrected by a 4-point solvent correction. For kinetic binding experiments, processed data were fitted to a 1:1 interaction model using the Biacore Insight Evaluation Software (version 3.0.12.15655).

#### Cell Culture

Immortalized murine bone marrow-derived macrophages were used (De Nardo et al., 2018). Cells were cultured in T-75 flasks with complete Dulbecco's modified Eagle's medium (DMEM) supplemented with 10% fetal bovine serum (FBS) and 1% penicillin-streptomycin in a humidified incubator at 37°C with 5% CO_2_.

#### Cell Viability Assay

NLRP3 KO immortalized murine bone marrow-derived macrophages overexpressing mouse NLRP3-FLAG and human ASC-mCerulean were seeded in 96 well plates and incubated overnight. Cells were treated with the fluorescent probes (**1** and **2**) and the biotin-tagged probes (**3** and **4**) at different concentrations in Opti-MEM for 4 h. Subsequently, cell viability was assessed using CellTiter-Blue^®^ cell viability assay according to the manufacturer's instructions.

#### Inflammasome and LDH Release Assays

NLRP3 KO Immortalized murine bone marrow-derived macrophages overexpressing mouse NLRP3-Flag and human ASC-mCerulean were seeded in 96-well plates and incubated for 16 h. Cells were treated with 400 ng/mL ultrapure LPS for 3 h. After priming, the probes or CRID3/MCC950 at various concentrations or DMSO were added to the cells for 30 min. Subsequently, to induce NLRP3 inflammasome activation, cells were treated with 10 μM nigericin for 1.5 h. As controls, to activate the AIM2 inflammasome, cells were transfected with 200 ng of poly(dA:dT) using 0.5 μL lipofectamine 2,000 per well for 4 h. Supernatants were collected to measure cytosolic LDH release by an LDH assay and IL-1ß release by Homogeneous Time Resolved Fluorescence (HTRF). All assays were performed according to the manufacturer's instructions. A two-tailed unpaired *t*-test was performed using GraphPad Prism 9.

#### Confocal Microscopy

Immortalized murine bone marrow-derived macrophages overexpressing NLRP3-mCitrine or mCitrine were seeded in Ibidi μ-slides. After 16 h, the fluorescent probe **2** was diluted in complete DMEM and added to the cells at 5 μM concentration for 30 min. Cells were fixed with 4% methanol-free formaldehyde for 10 min at room temperature. They were then washed three times with PBS and directly imaged with a Leica SP8 lightning confocal microscope.

## Data Availability Statement

The original contributions presented in the study are included in the article/Supplementary Material, further inquiries can be directed to the corresponding author.

## Author Contributions

TK and MGü designed compounds and wrote the manuscript. TK synthesized compounds. KG and MM performed SPR experiments. AA and ML performed cellular assays and microscopy. All authors analyzed data. MGe, EL, and MGü supervised the study. TK and KG contributed equally. MGü conceived the study.

## Conflict of Interest

The authors declare that the research was conducted in the absence of any commercial or financial relationships that could be construed as a potential conflict of interest.
