# Supplementary material for: Development of Fluorescent and Biotin Probes Targeting NLRP3
Source: Front Chem. 2021 Apr 22;9:642273. doi: 10.3389/fchem.2021.642273 (PMC8115122; doi:10.3389/fchem.2021.642273)
Supplement: Supplementary file 1 [file Data_Sheet_1.PDF]

Supplementary Material

SUPPLEMENTARY DATA

Syntheses

N-(4-(N-((1,2,3,5,6,7-hexahydro-s-indacen-4-yl)carbamoyl)sulfamoyl)benzyl)-11-oxo-2,3,6,7-tetrahydro-1H,5H,11H-pyrano[2,3-f]pyrido[3,2,1-ij]quinoline-10-carboxamide (**2**). The Boc-protected compound *tert*-butyl (4-(*N*-((1,2,3,5,6,7-hexahydro-*s*-indacen-4-yl)carbamoyl)sulfamoyl)benzyl)carbamate (**9**, 0.24 g, 0.5 mmol) was dissolved in dry CH_2Cl_2 (5 mL) and TFA (5 mL) was added. The mixture was stirred at room temperature for 2 h. After removal of the volatiles, the residue was further dried under high vacuum to yield the trifluoroacetate salt **10**. Compound **17** (0.14 g, 0.5 mmol) was dissolved in dry DMF (5 mL) under argon atmosphere. HATU (0.18 g, 0.55 mmol) and DIPEA (0.19 g, 1.5 mmol) were added and it was stirred for 30 min. Then, the entire material of the trifluoroacetate salt **10** was added. The mixture was stirred for 4 h at room temperature, was quenched with H_2O (20 mL), diluted with saturated NH_4Cl solution (50 mL) and extracted with EtOAc (3 × 30 mL). The combined organic phases were washed with sat. NH_4Cl (60 mL), dried over Na_2SO_4, filtered and concentrated. The crude product was purified via silica gel column chromatography using CH_2Cl_2/MeOH (19+1) as eluent. The product fractions were evaporated. The solid residue was resuspended in EtOAc, filtered, washed with MeOH and dried under high vacuum to yield a fine yellow solid. Yield 46%; mp: >250 °C (decomposition); R_f = 0.47 (CH_2Cl_2/MeOH 19+1); ^{1}H NMR (600 MHz, DMSO-d_6) δ 1.85 – 1.92 (m, 8H), 2.51 – 2.54 (m, 4H, signal is mainly obscured by DMSO signal), 2.69 – 2.71 (m, 2H), 2.72 – 2.76 (m, 6H), 3.31 – 3.34 (m, 4H, signal is mainly obscured by H_2O signal), 4.60 (d, *J* = 6.2 Hz, 2H), 6.91 (s, 1H), 7.23 (s, 1H), 7.53 (d, *J* = 8.0 Hz, 2H), 7.89 (d, *J* = 8.0 Hz, 2H), 8.06 (s, 1H), 8.51 (s, 1H), 9.17 (t, *J* = 6.1 Hz, 1H), 10.70 (s, 1H); ^{13}C NMR (151 MHz, DMSO-d_6) δ 19.55, 20.49 (two carbons), 24.95, 26.76, 30.03, 32.38, 42.15, 48.99, 49.52, 104.60, 107.36, 107.64, 117.94, 119.43, 127.15, 127.34, 127.60, 128.56, 137.14, 138.42, 143.02, 145.33, 147.72, 148.07, 149.00, 152.12, 161.78, 162.80; LC-MS (ESI) (90% H_2O to 100% MeOH in 10 min, then 100% MeOH to 20 min, DAD 220-500 nm), t_R= 11.81 min, 96% purity, *m/z* $[M+H]^+$ calcd for $C_{36}H_{36}N_4O_6S$ 653.2, found 653.5; HRMS (ESI) *m/z* $[M+Na]^+$ calcd 675.2248, found 675.2264.

N-(4-(N-((1,2,3,5,6,7-hexahydro-s-indacen-4-yl)carbamoyl)sulfamoyl)benzyl)-5-((3aR,4R,6aS)-2-oxohexahydro-1H-thieno[3,4-d]imidazol-4-yl)pentanamide (**4**). *tert*-Butyl (4-(*N*-((1,2,3,5,6,7-hexahydro-*s*-indacen-4-yl)carbamoyl)sulfamoyl)benzyl)carbamate (**9**, 0.24 g, 0.5 mmol) was dissolved in dry CH_2Cl_2 (5 mL) and treated with TFA (5 mL). The solution was stirred for 2 h at room temperature. Then it was evaporated and dried under high vacuum to give the trifluoroacetate salt **10**. Compound **20** (0.17 g, 0.5 mmol) was dissolved in dry DMF (5 mL). TEA (0.15 g, 1.5 mmol) and a solution of the trifluoroacetate salt **10** in DMF (5 mL) were added. The reaction mixture was allowed to stir overnight at room temperature. The solution was concentrated under high vacuum. The crude product was purified via silica gel column chromatography using CH_2Cl_2/MeOH (9+1) as eluent to yield a white solid. Yield 43%; mp: >230 °C (decomposition); R_f = 0.46 (CH_2Cl_2/MeOH 9+1); LC-MS(ESI) (90% H_2O to 100% MeOH in 10 min, then 100% MeOH to 20 min, DAD 220-400 nm), t_R= 10,05 min, 96% purity, *m/z* $[M+H]^+$ calcd for $C_{30}H_{37}N_5O_5S_2$ 612.2, found 612.6; HRMS (ESI) *m/z* $[M+H]^+$ calcd 612.2309, found 612.2303.

Benzyl (2-(2-hydroxyethoxy)ethyl)carbamate (**12**) (Adamczyk et al., 2002). 2-(2-Aminoethoxy)ethanol (**11**, 7.89 g, 75 mmol) was dissolved in dry CH_2Cl_2 (75 mL). TEA (7.68 g, 75 mmol) was added and the reaction mixture was cooled to 0 °C. Benzyl chloroformate (CAB; 12.18 g, 75 mmol) was dissolved in dry CH_2Cl_2 (75 mL) and was slowly added. It was stirred for 2 h at 0 °C and then for 18 h at room temperature. The reaction was quenched with saturated sodium bicarbonate solution (150 mL) and the aqueous layer was extracted with CH_2Cl_2 (3 × 150 mL). The combined organic layers were washed with brine (50 mL), dried over Na_2SO_4, filtered and concentrated. The crude product was purified via silica gel column chromatography using EtOAc as eluent to yield a colorless oil. Yield 88%; R_f = 0.48 (EtOAc); ^{1}H NMR (600 MHz, DMSO-d_6) δ 3.16 (q, *J* = 5.9 Hz, 2H), 3.39 – 3.42 (m, 4H), 3.48 (q, *J* = 5.3 Hz, 2H), 4.58 (t, *J* = 5.5 Hz, 1H), 5.01 (s, 2H), 7.27 (t, *J* = 5.8 Hz, 1H), 7.30 – 7.38 (m, 5H); ^{13}C NMR (151 MHz, DMSO-d_6) δ 40.27, 60.20, 65.24, 69.10, 72.11, 127.77, 127.78, 128.37, 137.21, 156.19; LC-MS (ESI) (90% H_2O to 100% MeOH in 10 min, then 100% MeOH to 20 min, DAD 200-400 nm), t_R= 8.37 min, 100% purity, *m/z* $[M+H]^+$ calcd for $C_{12}H_{17}NO_4$ 240.1, found 239.9.

tert-Butyl 3-oxo-1-phenyl-2,7,10-trioxa-4-azadodecan-12-oate (**13**) (Adamczyk et al., 2002). Compound **12** (1.20 g, 5 mmol) was dissolved in dry THF (10 mL) and cooled to 0 °C. A suspension of potassium *tert*-butoxide (0.56 g, 5 mmol) in dry THF (10 mL) was added. The reaction mixture stirred for 30 min at 0 °C. Then *tert*-butyl bromoacetate (1.17 g, 6 mmol) was added in one portion.

The solution was stirred for 3 h at 0 °C and then for 18 h at room temperature. H_2O (5 mL) was added and the mixture was concentrated. The residue was partitioned between EtOAc (50 mL) and H_2O (50 mL) and the aqueous layer was extracted with EtOAc (2×50 mL), dried over Na_2SO_4, filtered and concentrated. The crude product was purified via silica gel column chromatography using petroleum ether/EtOAc (2+1) as eluent to yield a colorless oil. Yield 57%; R_f = 0,33 (petroleum ether/EtOAc 2+1); ^{1}H NMR (600 MHz, DMSO-d_6) δ 1.42 (s, 9H), 3.15 (q, *J* = 5.9 Hz, 2H), 3.42 (t, *J* = 6.0 Hz, 2H), 3.50 – 3.57 (m, 4H), 3.97 (s, 2H), 5.01 (s, 2H), 7.24 (t, *J* = 5.7 Hz, 1H), 7.29 – 7.38 (m, 5H); ^{13}C NMR (151 MHz, DMSO-d_6) δ 27.73, 40.14, 65.18, 68.11, 69.07, 69.42, 69.82, 80.61, 127.68, 127.71, 128.30, 137.17, 156.13, 169.34; LC-MS (ESI) (90% H_2O to 100% MeOH in 10 min, then 100% MeOH to 20 min, DAD 200-400 nm), t_R= 10.85 min, 90% purity, *m/z* $[M+H]^+$ calcd for $C_{18}H_{27}NO_6$ 354.2, found 354.1.

11-Oxo-2,3,6,7-tetrahydro-1H,5H,11H-pyrano[2,3-f]pyrido[3,2,1-ij]quinoline-10-carboxylic acid (**17**). Isopropylidene malonate (**15**, 3.53 g, 10 mmol) and piperidinium acetate (6.44 g, 0.4 mmol) were added to a solution of 8-hydroxyjulolidine-9-carboxaldehyde (**16**, 4.47 g, 10 mmol) in absolute ethanol (10 mL). The mixture was allowed to stir at room temperature for 20 min and was then heated to reflux for 2 h. The reaction mixture was allowed to cool to room temperature and stored at –18 °C for 2 h. The crystallized solid was filtered off and washed with ice-cooled absolute ethanol (10 mL) to yield a pure orange solid. Yield 41%; mp: 250-252 °C, lit. mp: 253 °C (van Gompel and Schuster, 1987); R_f = 0.22 (petroleum ether/EtOAc 1+1 + 1% AcOH); ^{1}H NMR (600 MHz, DMSO-d_6) δ 1.84 – 1.92 (m, 4H), 2.69 – 2.75 (m, 4H), 3.33 – 3.37 (m, 4H), 7.24 (s, 1H), 8.45 (s, 1H), 12.41 (s, 1H); ^{13}C NMR (151 MHz, DMSO-d_6) δ 19.47 (two carbons), 20.44, 26.73, 49.09, 49.62, 104.71, 105.06, 107.22, 119.51, 127.45, 148.69, 149.15, 152.63, 160.55, 164.59; LC-MS (ESI) (90% H_2O + 0.1% AcOH to 100% MeOH + 0.1% AcOH in 10 min, then 100% MeOH + 0.1% AcOH to 20 min, DAD 220-500 nm), t_R= 10.43 min, 99% purity, *m/z* $[M+H]^+$ calcd for $C_{16}H_{15}NO_4$ 286.1, found 285.9 $[M+H]^+$.

2,5-Dioxopyrrolidin-1-yl 5-((3aR,4R,6aS)-2-oxohexahydro-1H-thieno[3,4-d]imidazol-4-yl)pentanoate (**20**). D-Biotin (**19**, 2.44 g, 10 mmol) was dissolved in dry DMF (70 mL) at 70 °C. Then it was allowed to cool to room temperature. DCC (2.06 g, 10 mmol) and pyridine (0.79 g, 10 mmol) were added to the solution and it was stirred for 5 min, before *N*-hydroxysuccinimide (1.50 g, 13 mmol) was added. The reaction mixture was stirred for 18 h at room temperature. The reaction mixture was filtered and concentrated under high vacuum. The residue was dispersed in isopropanol (150 mL) and refluxed for 30 min. The mixture was stored at –18 °C overnight. The precipitate

formed was filtered off and dried under high vacuum to yield a white solid. Yield 85%; mp: 198-200 °C, lit. mp: 199-201 °C (Piggott and Karuso, 2008); R_f = 0.41 (CH_2Cl_2/MeOH 9+1); ^{1}H NMR (500 MHz, DMSO-d_6) δ 1.38 – 1.46 (m, 2H), 1.46 – 1.55 (m, 1H), 1.60 – 1.70 (m, 3H), 2.59 (d, J = 12.4 Hz, 1H), 2.67 (t, J = 7.4 Hz, 2H), 2.80 – 2.86 (m, 5H), 3.08 – 3.13 (m, 1H), 4.13 – 4.17 (m, 1H), 4.29 – 4.33 (m, 1H), 6.33 (s, 1H), 6.39 (s, 1H); ^{13}C NMR (126 MHz, DMSO-d_6) δ 24.26, 25.40, 27.52, 27.79, 29.97, 55.16, 59.14, 60.96, 162.62, 168.86, 170.18. One signal is missing (overlapping solvent peak); LC-MS (ESI) (90% H_2O to 100% MeCN in 10 min, then 100% MeCN to 20 min, DAD 220-400 nm), t_R= 5.79 min, m/z $[M+H]^+$ calcd for $C_{14}H_{19}N_3O_5S$ 342.1, found 342.2.

SUPPLEMENTARY FIGURES

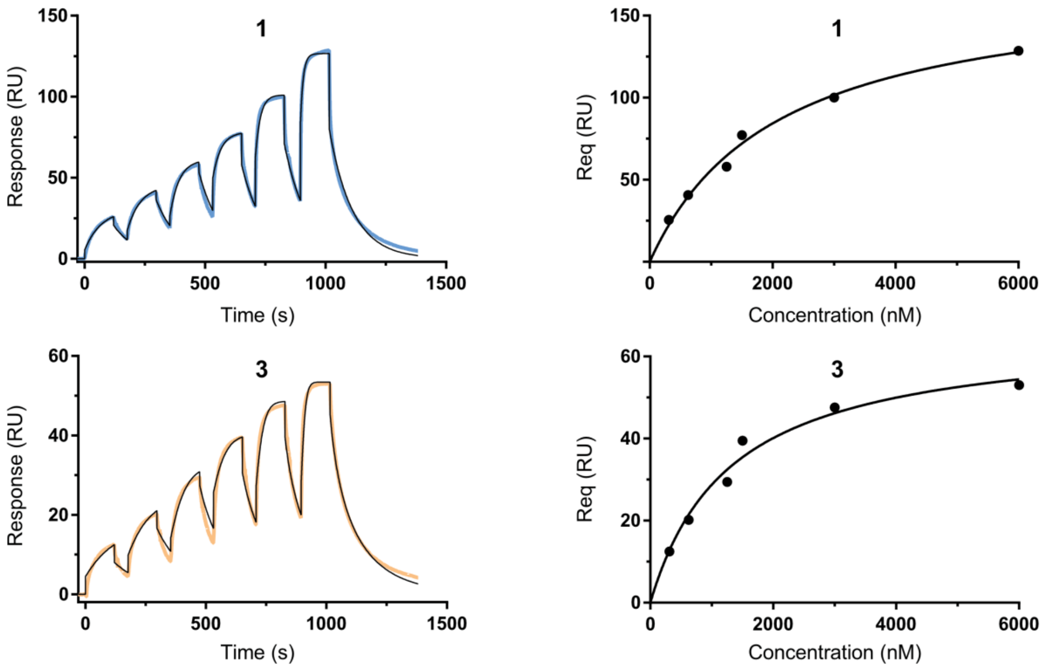

Figure S1. SPR sensorgrams (left) and concentration-binding response unit curves (right) of compound **1** (top) and compound **3** (bottom), injected on the chip surface loaded with human NLRP3-PYD-NACHT. The binding parameters obtained from the sensorgrams are listed in Table 1. Data were fitted to a 1:1 binding model.

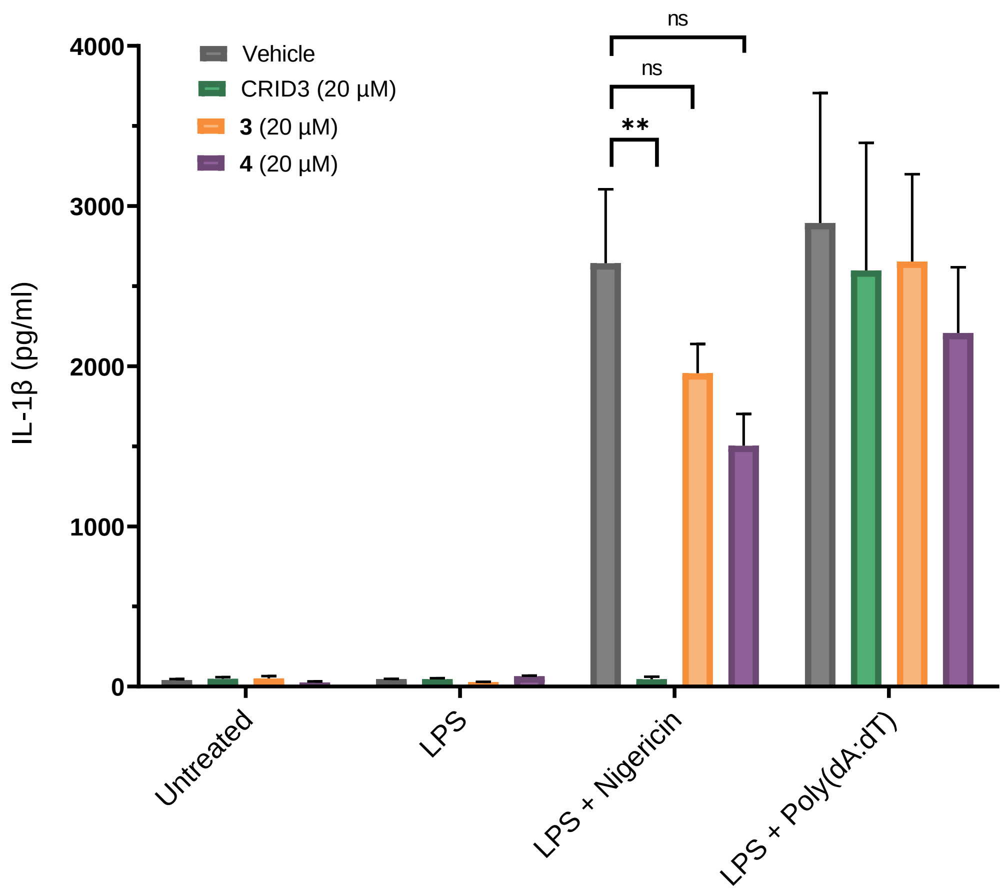

Figure S2. Inhibition of IL-1β release in bone marrow-derived macrophages after treatment with CRID3/MCC950 and biotin probes **3** and **4**. All data are means ± SEM (n=3), Student's t-test. * $P \leq 0.05$, ** $P \leq 0.01$, *** $P \leq 0.001$.

REFERENCES

Adamczyk, M., Fishpaugh, J. R., and Thiruvazhi, M. (2002). Concise synthesis of N-protected carboxyalkyl ether amines. *Org. Prep. Proced. Int.* 34, 326–331. doi: 10.1080/00304940209356773.

Piggott, A. M. and Karuso, P. (2008). Rapid identification of a protein binding partner for the marine natural product kahalalide F by using reverse chemical proteomics. *ChemBioChem* 9, 524–530. doi: 10.1002/cbic.200700608.

Van Gompel, J. and Schuster, G. B. (1987). Chemiluminescence of organic peroxides: Intramolecular electron-exchange luminescence from a secondary perester. *J. Org. Chem.* 52, 1465–1468. doi: 10.1021/jo00384a015.

frontiers
